# Regulation of Neurogenesis in Mouse Brain by HMGB1

**DOI:** 10.3390/cells9071714

**Published:** 2020-07-17

**Authors:** Xiang Zhao, Ari Rouhiainen, Zhilin Li, Su Guo, Heikki Rauvala

**Affiliations:** 1 Department of Bioengineering and Therapeutic Sciences, Programs in Human Genetics and Biological Sciences, Schools of Pharmacy and Medicine, University of California, San Francisco, CA 94143-2811, USA; su.guo@ucsf.edu; 2 Helsinki Institute of Life Science/Neuroscience Center, Biomedicum 1, P. O. Box 63 (Haartmaninkatu 8), University of Helsinki, FI-00014 Helsinki, Finland; ari.rouhiainen@helsinki.fi (A.R.); zhilin.li@helsinki.fi (Z.L.); 3 Translational Cancer Medicine, Research Programs Unit, Faculty of Medicine, University of Helsinki, FI-00014 Helsinki, Finland

**Keywords:** HMGB1, brain development, neurogenesis, differentiation, CXCL12, CXCR4

## Abstract

The High Mobility Group Box 1 (HMGB1) is the most abundant nuclear nonhistone protein that is involved in transcription regulation. In addition, HMGB1 has previously been found as an extracellularly acting protein enhancing neurite outgrowth in cultured neurons. Although HMGB1 is widely expressed in the developing central nervous system of vertebrates and invertebrates, its function in the developing mouse brain is poorly understood. Here, we have analyzed developmental defects of the HMGB1 null mouse forebrain, and further examined our findings in ex vivo brain cell cultures. We find that HMGB1 is required for the proliferation and differentiation of neuronal stem cells/progenitor cells. Enhanced apoptosis is also found in the neuronal cells lacking HMGB1. Moreover, HMGB1 depletion disrupts Wnt/β-catenin signaling and the expression of transcription factors in the developing cortex, including Foxg1, Tbr2, Emx2, and Lhx6. Finally, HMGB1 null mice display aberrant expression of CXCL12/CXCR4 and reduced RAGE signaling. In conclusion, HMGB1 plays a critical role in mammalian neurogenesis and brain development.

## 1. Introduction

The High Mobility Group Box 1 protein (HMGB1, alias is HMG1 and Amphoterin) is a conserved protein that is widely expressed in almost all types of cells from embryo to adulthood [1,2]. HMGB1 is not only the main parental member of the HMG protein family involved in intracellular regulation, but it also has extracellular regulatory functions that are related to development, disease, and inflammation.

Extracellular HMGB1 was initially found as a heparin-binding protein abundantly expressed in the embryonic rat brain [3,4]. HMGB1 secreted from neurons and from non-neuronal cells [5,6], mainly signals through the RAGE (Receptor for Advanced Glycation End products) receptor [7,8], and it plays an essential role in neurite outgrowth and neuronal migration in the developing nervous system [9]. In recent years, the extracellular functions of HMGB1 have been intensively investigated. HMGB1 is released by the immune cells and platelets under special stimuli, such as infection or injury, and it is crucial for cell migration, differentiation, and activation [10,11,12]. HMGB1 has been described as an alarmin or damage-associated molecular pattern (DAMP) molecule as for the central role of HMGB1 in inflammation and a relevant molecule in immune responses against tumor formation [13].

HMGB1 has been found to be highly expressed in the developing central nervous system (CNS) across different species [14,15,16,17]. Interestingly, HMGB1 shows a specific dynamic expression pattern in the embryonic mouse brain. It is highly expressed in the dorsal telencephalon until E16 (Embryonic day 16) and decreases dramatically after E18 [18], which suggests its involvement in neurogenesis during early development of the brain. When compared to pathophysiological roles of HMGB1 in the immune system, its role in the CNS is not well understood.

Recently, HMGB1 was found to form a HMGB1-CXCL12 heterocomplex that signals through CXCR4 in leukocytes [19]. HMGB1 and CXCL12/CXCR4 chemotaxis play an important role in recruiting immune cells to the site of inflammation. CXCL12/CXCR4 signaling regulates cerebellar granule cell development [20,21,22], neurogenesis, and neuronal migration in the developing CNS [23,24]. Furthermore, CXCL12/CXCR4 is critical for the development of the hippocampal dentate gyrus, the site of active adult neurogenesis [25]. CXCL12 expression increases at the site of apoptosis in the developing brain, followed by an increased presence of microglia at the apoptotic site [26]. HMGB1 is also found to be required for the microglia activation in the neuroinflammatory responses [27]. The high expression of HMGB1 and CXCL12/CXCR4 has been found to be coordinated in the prenatal cortex, while there is short of evidence of HMGB1 and CXCL12/CXCR4 signaling in the nervous system as compared to the immune system [28,29].

Our study has investigated HMGB1 in vivo function in the developing mammalian brain for the first time using the HMGB1 knockout (KO) mice. We have found dramatically reduced neurogenesis and differentiation in the developing brain of the HMGB1 KO mouse embryos. HMGB1 knockout mice showed prominent forebrain hypoplasia at early developmental stage. Such defects in the HMGB1 KO mouse embryos have been proven by aberrant expression of multiple neuro-developmental transcription factors shown by in situ and qRT-PCR analyses. Furtermore, we have clearly demonstrated that HMGB1 is required for maintaining CXCL12/CXCR4 expression in the developing brain by both in vivo and in vitro immunohistochemistry results. Our data provide the first evidence that the HMGB1 dependent CXCL12/CXCR4 signaling pathway also exists in the mammalian developing CNS.

## 2. Materials and Methods

### 2.1. Animals

Original strain of *Hmgb1*^+/−^ mice (CBA/C57BL background) has been kindly provided by Professor Marco Bianchi’s lab. The pleiotropy of *Hmgb1*^−/−^ phenotypic defects is consistent across different genetic backgrounds. The original *Hmgb1*^+/−^ male mice were bred to the CD-1 mouse strain in order to elevate their fertility. The *Hmgb1*^+/−^ hybrids were used for producing *Hmgb1*^−/−^ littermates. In breedings, the day of detection of the vaginal plug was determined as E0. HMGB1 knockout (KO) mouse genotyping has been done according to the previous description [30]. The animal experiment permits were obtained from the Office of the Regional Government of Southern Finland (license number ESAVI/6603/04.10.03/2011) in agreement with the ethical guidelines of the European convention. All experimental protocols were carried out in accordance with the approved guidelines of the Laboratory Animal Centre (LAC) of the University of Helsinki.

### 2.2. Bromodeoxyuridine (BrdU) Labeling of Neuronal Progenitors In Vivo

BrdU was dissolved in sterile saline and injected intraperitoneally into pregnant dams at the saturating dose of BrdU (50 μg/g body weight, two times injection with a 6-h interval) one day before collecting the embryos. Multiple injections were used to ensure the labeling of most cells undergoing cell divisions over the period of cell cycle in dorsal telencephalon [31,32]. Within 2 h after the successive BrdU injections, pregnant mice were sacrificed and E10 and E16 embryos were collected and then fixed in 4% paraformaldehyde by immersion and embedded in paraffin. Sections of 15 µm were cut and immunostaining was done with rat anti-BrdU antibody (ab6326, Abcam, Cambridge, MA) in 1:300 dilution after pretreatment of the tissue with 1 N hydrochloric acid for 15 min. at 37 °C, followed by 5 min. in borate buffer. Subsequently, 3,3′-Diaminobenzidine (DAB) colorimetric staining was used for immunodetection with horseradish peroxidase (HRP) conjugated secondary antibodies. In the end, the stained sections were mounted by UltraKitt™ (Biosystem Switzerland AG, Muttenz, Switzerland) mounting media after dehydration. For the WT and KO embryos, both sagittal and coronal sections were continuously collected with the interval of 60 μm (thickness of the section is 15 μm, one section was attached to the glass when cutting four sections off). All of the sections from the same embryo were clearly numbered by the sequence of collection. The sections of the WT and KO embryos were anatomically aligned by referring to Kaufman’s Atlas of Mouse Development and the Allen Developing Mouse Brain Atlas (2015).

### 2.3. In Situ Hybridizations and Histology

E16 embryo paraffin sections were prepared, as above. In situ RNA hybridization on dehydrated E16 embryo sections was performed in the InSitu ProVSi device (Intavis AG, Köln, Germany) [14]. Luca Muzio’s laboratory kindly offered Tbr2, Emx2, Foxg1, and CXCL12 cDNA for antisense and sense probes [26,33,34]. CXCR4 RNA probes were generated, as described [22]. Lhx6 cDNA fragment (TC1570995.1) was produced for synthesizing the RNA probe according to the information on the webpage of Allen Mouse Brain Atlas.

Mayer’s Hematoxylin (Thermo Fisher Scientific, Waltham, MA, USA) was used for nuclear counterstaining of E16 embryo sections following the recommended protocol. The colorimetrically stained sections were mounted by UltraKitt™ mounting media after dehydration.

### 2.4. qRT-PCR Array

Total RNA from E16 embryonic brain was extracted with NucleoSpin RNA kit (Macherey-Nagel GmbH & Co. KG, Düren, Germany). RNA was reverse-transcribed as described [14]. Tested genes were selected based on Mouse neurogenesis & differentiation RT^2^ Profiler™ PCR Array (Qiagen, Hilden, Germany). Tested qRT-PCR primers of *Ascl1, Bmp2, Bmp4, Hes1, Neurod1, Neurog1, Neurog2, Olig2, Sox2, Shh, Ntf3, Pax6, Notch2, Ache, Wnt1, Wnt3, Bdnf, Egf, Gdnf, Fgf2, Cxcl1, Tgfb1, Tbr2, Emx2, Apoe,* and *Bcl2* (including *β-Actin*, *Gapdh* and *Rpl13a* as reference genes) were ordered from Sigma-Aldrich (St. Louis, MO, USA). The *Cxcl12* primers were 5′-TGC ATC AGT GAC GGT AAA CCA -3′ and 5′-GTT GTT CTT CAG CCG TGC AA- 3′. *Cxcr4* primers were 5′- GAC TGG CAT AGT CGG CAA TG -3′ and 5′-AGA AGG GGA GTG TGA TGA CAA A- 3′. The *Cxcr7* primers were 5′-CCT AAC AAG AAC GTG CTT CTG T- 3′and 5′-GTG GTC TTA GCC TGG ATA TTC AC- 3′. The *Amigo1* primers were 5′-TCG GTG AGC GTG AGG AAT G -3′ and 5′- CCC ACC AGG GTA GTG TAG G-3′. The PCRs were processed with the Bio-Rad CFX96 real-time PCR machine using the *CFX96*™ real-time PCR detection system (Bio-Rad Laboratories Inc., Hercules, CA). The LuminoCt™ SYBR Green qPCR ReadyMix™ (Sigma-Aldrich) was used with the PCR protocol as before [14].

### 2.5. Fluorescence-Activated Cell Sorting (FACS)

Dissected embryonic E14.5 brains were titrated in FACS buffer (PBS - 1% Fetal calf serum - 0.02% NaN_3_) using pipette tip, and filtered through 70-µm cell strainers (Fisher Scientific, Hampton, NH). Single cell suspensions were blocked with 10% normal rat serum on ice for 30 min., and stained with a combination of anti-mouse cell surface flow cytometric markers CD11b-FITC (BioLegend, San Diego, CA), GLAST-PE, and O4-APC (Miltenyi Biotec, Bergish Gladbach, Germany) at 4 °C in dark for 1 h with continuous rotation. The stained cells were then washed, centrifuged, and resuspended into 1 mL of FACS buffer, and acquired on a two-laser, six-color Gallios cytometer (Beckman Coulter, Pasadena, CA). The threshold for FACS analysis was set based on the surface expression of each individual marker in the samples as compared to isotype control using IgG-stained cells in the same channel, and Flow cytometric data were analyzed with the Kaluza 1.3 software (Beckman Coulter, Brea, CA), as reported [35,36]. Cell populations were calculated as the percentages among the total cells.

### 2.6. Primary Neuronal Cultures

Primary cortical neurons were prepared from both E14 and E16 pup cortex. The cleared cell suspension containing 80–90% of neurons or neuronal precursors was collected and plated for the assays [5]. The cells were cultured in neurobasal medium (Thermo Fisher Scientific) with B27 supplement (Thermo Fisher Scientific) and penicillin–streptomycin and used at two, four, and seven days in vitro for treatments and/or immunocytochemistry. For each brain, the harvested cortical neurons were divided into four wells on the 24-wells plate that was coated with ploy-l-lysine and cultured with or without HMGB1 recombinant protein (recHMGB1). recHMGB1 was previously produced in animal cells and shown to correspond in its properties to HMGB1 isolated from rat brain [37]. For recHMGB1 supply in the cell culture, 20 µg/mL recHMGB1 were coated to the plate for 1 h at room temperature and the wells were then washed by culture medium. The suspended cells were added into the wells with equal volume (4–5 × 10^5^ cells) containing 10 µg/mL recHMGB1 [37,38]. For maintaining the concentration of recHMGB1 in the cell culture, the culture medium was replaced every 48 h by freshly prepared 10 µg/mL recHMGB1 in the cell culture medium. The cultures were incubated for 2, 4 or 7 days before fixation as defined in each experiment.

### 2.7. Antibodies, Western Blotting and Immuno-Cytochemistry

Affinity-purified Rabbit-anti-HMGB1 antibody (1 mg/mL) against the peptide KFKDPNAPKRPPSA that corresponds to the residues 87–100 of the rat HMGB1 sequence was used for Western blotting and fluorescent immunostaining (1:1000) [14,39]. Rabbit Anti-CXCR4 (Polyclonal, Abcam, Cambridge, UK; ab2074, 1 mg/mL, 1:400) and anti-CXCL12 (1 mg/mL; 1:200) were used for Western blotting and fluorescent immunostaining. Rabbit anti-PCNA antibody (1 mg/mL, Abcam, ab15497, 1:200) was used for fluorescent immunostaining. The Rabbit-anti-mouse AMIGO1 antibodies (1 mg/mL, Neuromab, Davis, CA; 28330) were used in western blotting (1:1000) and fluorescent immunostaining (1:500). Goat anti-RAGE (Fisher Scientific, AF1145SP) antibody was used for Western blotting (1 mg/mL, 1:2000). The rabbit anti-NeuN antibody (Invitrogen, PA5-78499), mouse anti-glial fibrillar acidic protein (GFAP) antibody (Fisher Scientific, 50-175-341) and mouse anti-β-Tubulin III (Tuj1) antibody (Sigma, T8578) were used for fluorescent immunostaining (1 mg/mL; 1:500). Anti-β-catenin antibodies (1 mg/mL, 1:400; C2206 Rabbit polyclonal, Sigma-Aldrich) were used for Western blotting and fluorescent immunostaining. Mouse monoclonal anti-β-actin antibody (A2228, 1:2000, Sigma-Aldrich) was used as a control of sample loading in Western blotting.

E16 mouse cortex was dissected and homogenized with rotor–stator homogenizer. The primary neuronal cells and tissue samples were lysed by SDS extraction buffer [40], and 15 μL of cell or tissue lysate was used for SDS-PAGE. Proteins were transferred to HybondTM nitrocellulose membrane (Amersham Biosciences, Little Chalfont, UK) by a semidry blotting technique with transblotting cell (Bio-Rad) and detected with the antibodies. After the HRP-conjugated secondary antibody incubation, the samples were visualized with the ECL Western blotting detection system (Amersham Biosciences).

For immunohistochemistry of E10, E12, E14, and E16 embryonic sections, the embryos at each developmental stage were collected. The torsos were used for genotyping. The dissected embryos were fixed in 4% PFA overnight at 4 °C. The fixed samples were processed by dehydration and paraffin embedding and cut into 10 µm sections. The rehydration and antigen retrieve of paraffin sections was done as introduced before [41]. The hydrated tissue sections and cell culture slides for immunostaining were then fixed in 4% PFA for 30 min. at room temperature. The sections were washed and preincubated in phosphate-buffered saline containing 0.1% Tween 20 (PBS-T, pH 7.4) and 4% normal goat serum at 4 °C overnight or longer. The specimens were incubated with the primary antibodies in the preincubation solution (PBS-T with 2% normal goat serum) for 3 h at room temperature. The samples were then washed thoroughly with PBS-T and incubated with the Alexa^®^-conjugated goat anti-rabbit (Alexa488), Donkey anti-goat (Alexa488 & Alexa546), or goat anti-mouse (Alexa568) secondary antibodies (diluted 1:2000) in the preincubation solution for 2 h at room temperature or overnight at 4 °C. The fluorometric TUNEL system (Alexa^®^ 488, DeadEndTM, Promega) was used for staining of apoptosis in whole mounts.

Immunocytochemistry of primary neurons was carried out, as described [41,42]. The stained neuronal cells were finally counter-stained with nuclear stain DAPI (Molecular Probes, Inc., Eugene, OR). The samples were washed with PBS-T twice for 15 min. each, three times with PBS for 10 min. each time, and covered by mounting medium (Fluoromount™ Aqueous, Sigma-Aldrich).

### 2.8. Imaging

Zeiss LSM 710 and LSM 780 confocal microscopy system (Carl Zeiss MicroImaging GmbH, Jena, Germany) were used for imaging embryo sections and cultured cell slides. Stacks of images that were taken at 1 µm intervals were compiled to make maximum intensity projection images. The specimens from in situ hybridization were examined with inverted light microscopy using Olympus IX 70 that was connected through a CCD camera to the Analysis^®^ software (Olympus, Tokyo, Japan). High resolution images were obtained with a digital MicroFire S99808 camera (Optronics, Goleta, CA) that was attached to Olympus BX51 epifluorescence microscope (Olympus). The acquired images were further processed with CorelDRAW^®^ Graphics Suite X6 (Corel Corporation, Ottawa, Canada).

### 2.9. Image Analyses

Confocal images of cell cultures were processed by Zeiss Efficient Navigation (ZEN 2012) software (Carl Zeiss MicroImaging GmbH) with single maximum intensity z-stacking projections containing the whole image stacks. The intensities of the Western blotting bands were examined with Quantity One version 4.6.2 (Bio-Rad). The measurement of BrdU and Hematoxylin staining in dorsal telencephalon were carried out using Fiji (ImageJ Version: 2.0.0-rc-69/1.52p). The BrdU staining cells and DAPI staining nucleus were counted by “Analyze Particles” build-in of Fiji. The anti-HMGB1, anti-PCNA antibody staining cells, and Tunel staining cells were counted by “Cell Counter” plug-in of Fiji. The collected primary neuronal cells from each brain were equally distributed and then cultured in three wells for three independent analyses. The average of the three wells was used for statistical analyses in groups.

### 2.10. Statistical Analyses

Unpaired *t*-test (two-tailed) has been carried out using OriginPro version 9.0. (OriginLab Corporation, Northampton, MA). Measurements are given in mean ± Standard deviation (SD) and Standard error mean (SEM), unless stated otherwise.

## 3. Results

### 3.1. Depletion of HMGB1 Results in Severe Defects in Brain Morphogenesis

HMGB1 KO mice were produced each time by dihybrid cross from HMGB1 heterozygous parents. HMGB1 heterozygous mice do not have prominent abnormalities and they produce offsprings normally [30]. The mouse embryos at E10 and E16 stage were used for the comparison of dorsal telencephalon between the HMGB1 KO and the WT embryos. All of the mouse embryos were collected from the uterus after careful plugging. Strikingly, the HMGB1 KO embryos showed significant morphogenesis defects.

**Figure 1:**
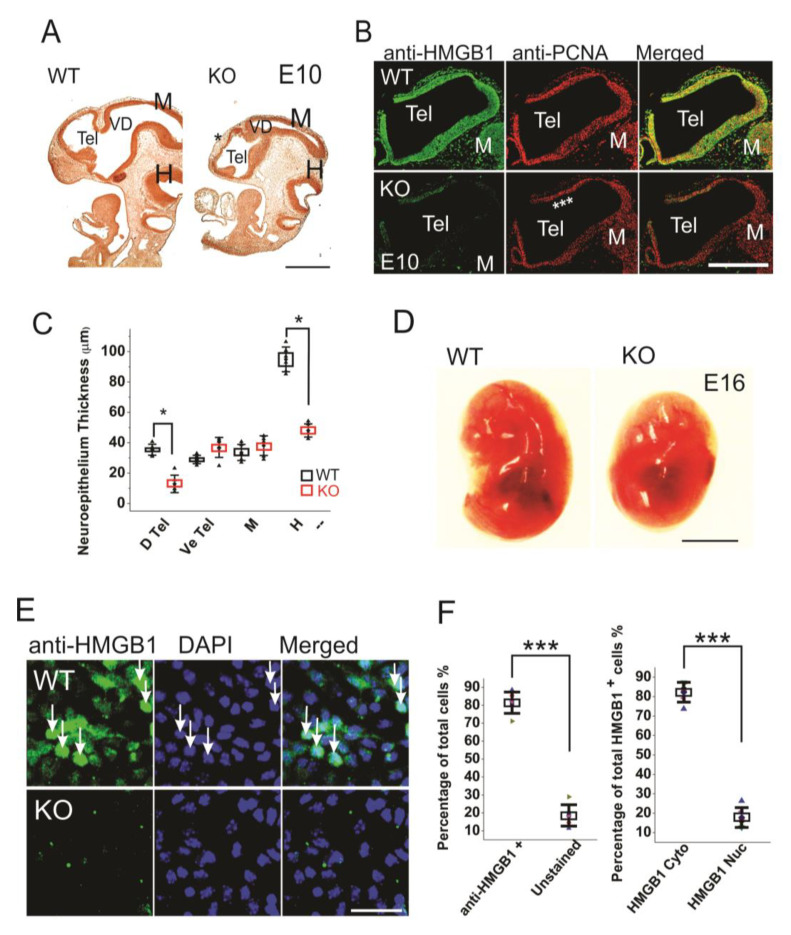
Morphogenesis and proliferation defects of the prenatal HMGB1 knockout (KO) mouse. (**A**) Bromodeoxyuridine (BrdU) stained sagittal sections of the E10 embryo from the HMGB1 KO and the WT control. The decreased BrdU staining in the dorsal neopallial cortex is indicated with *. Scale bar indicates 500 µm. (**B**) Whole mount immunostaining of sagittal sections of E10 dorsal telencephalon with anti-HMGB1 (Green) and anti-PCNA (Red) antibodies. HMGB1 was not detected in the HMGB1 KO. PCNA staining was grossly decreased in the rostral anterior part of the dorsal telencephalon (indicated with ***). Scale bar indicates 400 µm. (**C**) Statistics of the thickness of E10 neuroepithelium. The BrdU stained sections from six KO and six WT embryos were compared. The HMGB1 KO showed significantly decreased BrdU staining in dorsal telencephalic ventricle and hindbrain. D Tel, dorsal telencephalic ventricle; Ve Tel, ventral telencephalic ventricle; M, midbrain; H, hindbrain. Mean values ± S.D. (error bars) and S.E.M (Boxes) are indicated (*n* = 6; * *p* < 0.001, unpaired *t*-test, two-tailed). (**D**) Lateral view of the E16 KO and the WT embryos. Scale bar indicates 5 mm. (**E**) Immunofluorescent staining of in vitro one day cultured E16 cortical neurons with anti-HMGB1 antibody (Green). Arrows show cells with high HMGB1 expression in nucleus. Scale bar indicates 20 μm. (**F**). The statistics of HMGB1 expressing cells in cultured E16 cortical neurons. The cortical neurons from five KO and five WT E16 brains were collected. Mean values ± S.D. (error bars) are indicated (*n* = 5; *** *p* < 0.001, unpaired *t*-test, two-tailed).

When compared to the WT embryos, the HMGB1 KO embryos are much smaller, and the defects in brain development can be clearly seen by BrdU staining already from E10 stage (Figure 1A). The depletion of HMGB1 expression in HMGB1 KO embryos was confirmed by the immunostaining of E10 sections and primary neuronal culture of E16 developing cortex with anti-HMGB1 antibody (Figure 1B,E). The HMGB1 KO had gross defects in Tel (telencephalic vesicle), which did not show the normal expansion towards dorsal-rostral direction as in the WT control (Figure 1A). The HMGB1 KO also showed decreased anti-PCNA staining in the dorsal telencephalon as compared to WT at E10 (Figure 1B). Notably, the neuroepithelial thickness of the dorsal pallium and hindbrain in the HMGB1 KO decreased dramatically when compared to the WT at E10 (Figure 1C). At E16, the morphological difference between the KO and the WT was even more clear (Figure 1D). HMGB1 expression was detected in over 80% of neuronal cells in the primary neuronal culture from the E16 WT embryos, but not from the KO embryos (Figure 1E,F). Only ~20% of HMGB1 expressing cells showed much higher HMGB1 expression in the nucleus than in the cytoplasm (Figure 1F).

**Figure 2:**
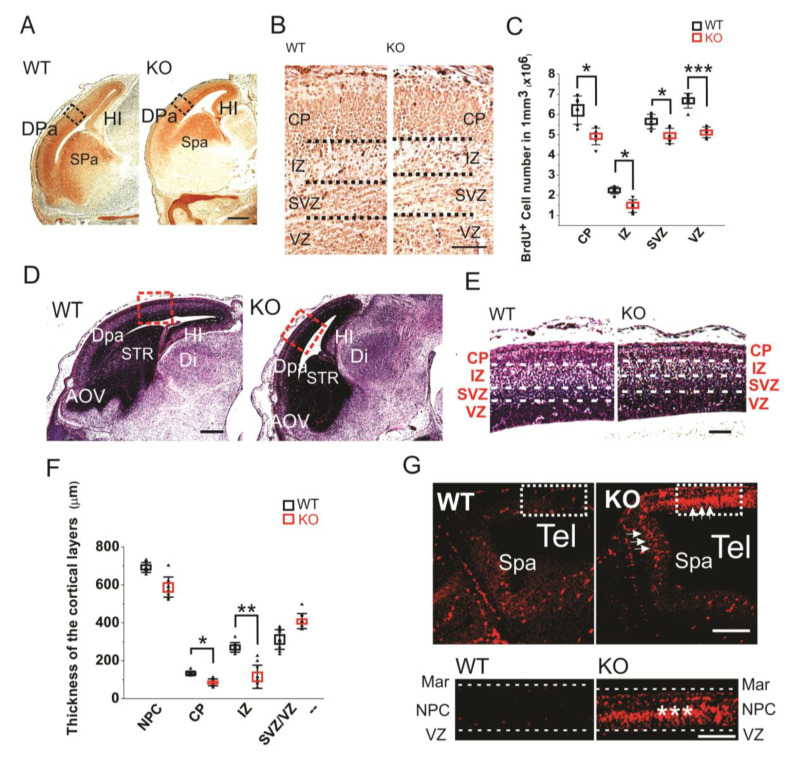
(**A**) Sagittal sections of E16 embryos with BrdU staining. Scale bar indicates 500 µm. The black rectangles are the areas shown in B. (**B**) BrdU staining of E16 dorsal telencephalon under higher resolution. Scale bar indicates 100 µm. (**C**) Statistics of the BrdU^+^ cell numbers in the E16 cortical layers of the KO and WT embryos. For each brain, BrdU^+^ cell numbers in each layer were counted five times at different sections for averaging. The stained sections from 5 KO and 5 WT embryos were used for comparison. Mean values ± S.D. (error bars) and S.E.M (Boxes) are indicated (*n* = 5; *** *p* < 0.001; * *p* < 0.05, unpaired *t*-test, two-tailed). (**D**) Sagittal sections of E16 embryos with hematoxylin staining. The areas in red rectangle were zoomed out in (**E**) Scale bar indicates 500 µm. (**E**) The E16 cortical layers in the dorsal pallium (Dpa) with hematoxylin stains. Scale bar indicates 150 µm. (**F**) Statistics of the thickness of E16 cortical layers. Each layer has been measured on three different sections at central position for each embryo. Sections from six KO and six WT mice have been measured. Mean values ± S.D. (error bars) and S.E.M (Boxes) are indicated (*n* = 6; **, *p* < 0.01; *, *p* < 0.05, unpaired *t*-test, two-tailed). (**G**) The Tunel staining of E12 sagittal sections. The strong apoptotic signaling in the dorsal neopallial cortex (NPC) and subpallium (Spa) of the HMGB1 KO is indicated by white arrows. The area in the white rectangle has been amplified. The intermediate layer of NPC showed highest apoptotic activity (***). Scale bar indicates 200 µm in the upper panel and 100 µm in the lower panel. Abbreviations: AOV, ventral anterior olfactory area; CP, cortical plate; Dpa, dorsal pallium/isocortex; H, hindbrain; HI, hippocampus; IZ, intermediate zone; M, midbrain; MAR, marginal layer; NPC, neopallial cortex; Spa, subpallium; STR, striatum; SVZ, subventricular zone; Tel, telencephalic vesicle; VD, ventricles of diencephalon; VZ, ventricular zone.

The brain developmental defects were further investigated by BrdU labeling of the sagittal sections of E16 embryos (Figure 2A,B). The BrdU labeling of E16 sections clearly showed that the HMGB1 KO embryos had less proliferating neuronal cells in dorsal telencephalon than the WT embryos (Figure 2A). The HMGB1 KO mice showed significantly decreased numbers of BrdU^+^ cells in the cortical layers under higher magnification (Figure 2B). As compared to the WT mice, the HMGB1 KO mice showed over 40% decreased numbers of BrdU^+^ cells in the intermediate zone (IZ), about 15–30% decrease in the subventricular zone (SVZ) and ventricular zone (VZ), and about 20% decrease in the cortical plate (CP) (Figure 2C).

The hematoxylin staining of E16 sections confirmed the prominent defects of prenatal forebrain development in the HMGB1 KO embryos with decreased staining in dorsal telencephalon as compared to the WT embryos (Figure 2D). The HMGB1 KO mice had much shorter length of the developing cortex in the direction from rostral to caudal than the WT (Figure 2D). Additionally, HMGB1 KO mice showed significantly decreased thickness of CP (Figure 2E). Furtermore, we found that the HMGB1 KO mice had much thinner IZ layer (less than 50%) than the WT at E16 (Figure 2E). In contrast, the thickness of the layer containing both SVZ and VZ (SVZ/VZ) in the HMGB1 KO mice did not show significant difference when compared to the WT mice (Figure 2F).

Furthermore, we found that the HMGB1 KO had dramatically upregulated apoptosis in the dorsal neopallial cortex (NPC) between the ventricular zone (VZ) and the marginal layer (MAR) at E12 (Figure 2G). In contrast, very limited numbers of apoptotic cells were found in the dorsal NPC of the WT control (Figure 2G). This difference explains the decreased thickness of dorsal telencephalon of HMGB1 KO embryos.

Taken together, HMGB1 depletion results in the downregulation of neurogenesis and upregulation of neuronal apoptosis in the developing mouse brain, which causes the severe hypoplasia of the HMGB1 KO mouse brain.

### 3.2. Induced Apoptosis and Reduced Neurogenesis in HMGB1 KO Neuronal Cells

We examined apoptosis, proliferation, and differentiation of cultured cells derived from the E16 dorsal telencephalon in order to understand the mechanisms underlying the impaired development of prenatal cortex found in the HMGB1 KO mice.

**Figure 3:**
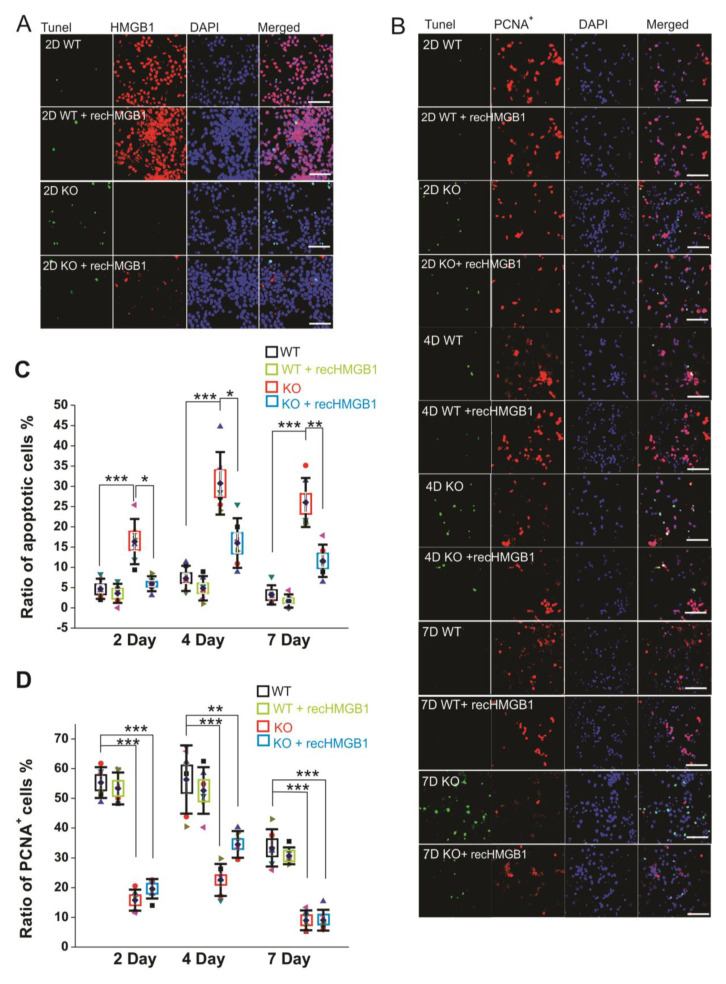
Apoptotic and proliferating activity of in vitro cultured E16 cortical neuronal cells. (**A**) Tunel staining of E16 cortical neuronal cells cultured for 2 days. All of the samples have been co-stained with anti-HMGB1 antibody (Alexa 568) and DAPI. Scale bar indicates 40 μm. (**B**) Tunel staining and anti-PCNA antibody (Alexa 568) staining of E16 cortical neuronal cells cultured for two, four, and seven days. Cell nuclei have been stained with DAPI. Recombinant HMGB1 protein (recHMGB1) was coated on the matrix and added into the culture medium at 10 μg/mL. Scale bar indicates 50 μm. (**A**) and (**B**) are the confocal z-stack max projections of 10 μm. (**C**) The statistics of apoptotic cells in each group of cultured cells shown in (**B**). (**D**) The statistics of proliferating cells in each group of cultured cells shown in (**B**). For the statistics in (**C**) and (**D**), the cortical neurons from six KO and six WT E16 brains were collected and cultured for the immunostaining and analysis. For each brain sample, three repeats were applied. Mean values ± S.D. (error bars) and S.E.M (Boxes) are indicated (*n* = 6; *** *p* < 0.001; ** *p* < 0.01; * *p* < 0.05; unpaired *t*-test, two-tailed).

After culturing of 2 days, there were more apoptotic cells in the HMGB1 KO cell culture than in the WT cell culture (Figure 3A). The apoptosis in the HMGB1 KO cells after culturing for four days and seven days was higher than in WT cells after culturing only for two days (Figure 3B). The apoptotic level of the cell cultures was analyzed with Tunel fluorescent staining (Figure 3C). In the HMGB1 KO cells cultured for two days, there were over 10% apoptotic cells. In four days and seven days, the apoptotic cells in the HMGB1 KO cultures were about 30% of the cell population (Figure 3C). The WT cell culture showed very low apoptosis at these time points (Figure 3A,B). We found the apoptosis of the HMGB1 KO cells to be dramatically decreased by the addition of exogenous recombinant HMGB1 (10 μg, but the WT cells were not affected by adding recombinant HMGB1 (Figure 3C).

We also examined the proliferation of cultured cells by immunostaining with anti-PCNA antibody (Figure 3B). The HMGB1 KO cells showed much less proliferation than the WT cells. About 80% of the WT cells showed proliferating activity after being cultured for two days and four days, and over 60% of the WT cells were actively proliferating after seven days (Figure 3D). In the HMGB1 KO cells cultured for two days and four days, there were only about 40–50% proliferating cells. The proliferation was more significantly decreased in the KO cells than in the WT control cells after culturing for seven days, with only about 5% of cells actively proliferating (Figure 3D). The recombinant HMGB1 added into the culture medium did not rescue the proliferating activity in the HMGB1 KO cells (Figure 3B,D).

Our results show that the HMGB1 KO cells display reduced neurogenesis and increased apoptosis. After adding recombinant HMGB1 protein, the apoptosis of HMGB1 KO cells was significantly attenuated, but the proliferation was not restored.

### 3.3. HMGB1 Depletion Downregulates Neural Differentiation

The HMGB1 KO embryos showed defective brain with grossly decreased hematoxylin staining of neuronal cells in the prenatal cortex as compared with the WT (Figure 2). This suggested that HMGB1 may have a role in neuronal differentiation.

**Figure 4:**
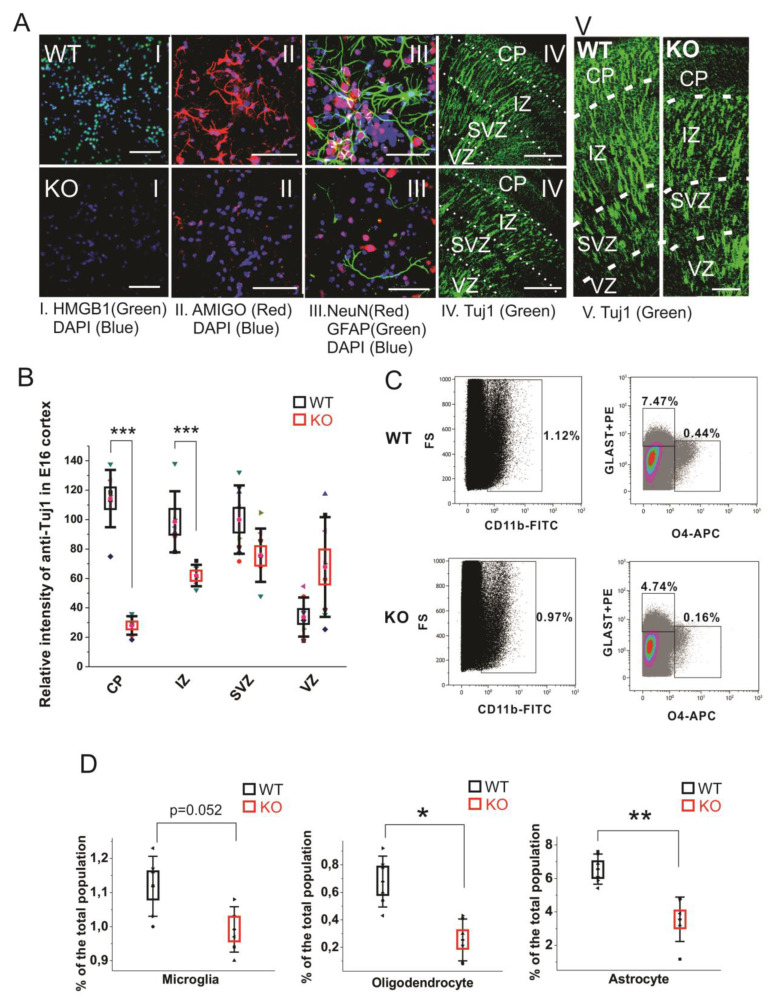
Cell differentiation activity in the WT and HMGB1 KO. (**A**) E16 cortical neuronal cells cultured for 7 days. (A I) Anti-HMGB1 staining (Green). (A II) Anti-AMIGO1 staining (Red). (A III) Anti-GFAP (Green) and anti-NeuN (Red) staining. DAPI (Blue) was used for labeling cell nuclei in (A I–III) Scale bar indicates 50μm. IV, Anti-β-Tubulin III (Tuj1) staining of E16 sagittal sections. Scale bar indicates 400 μm. (A V) Anti-β-Tubulin III (Tuj1) staining of E16 sagittal sections. Scale bar indicates 100 μm. (I–V) are the confocal z-stack max projections of 10μm. (**B**) Statistics of Tuj1 fluorescent staining intensity in the WT and HMGB1 E16 dorsal telencephalon. The HMGB1 KO showed significantly decreased Tuj1 staining in CP and IZ layers compared to the WT. The HMGB1 KO showed higher Tuj1 staining in VZ than the WT. Statistics has been done by the six brain sections from three embryos for both WT and KO. Mean values ± S.D. (error bars) and S.E.M (boxes) are indicated (*n* = 6; *** *p* < 0.001, unpaired *t*-test, two-tailed). (**C**) Flow cytometry analyses of CD11b^+^ microglia cells, GLAST-PE^+^ astrocytes, and O4-APC^+^ oligodendrocytes in the E16 WT or HMGB1 KO brains. Each sample was prepared from one single brain. The ratio of each type of cells among the total cell population was then calculated. (**D**) Statistics analysis of Flow cytometry results of the WT and KO mouse brains. The HMGB1 KO mouse brain showed significantly reduced population of oligodendrocytes and astrocytes compared to the WT controls. Statistics is based on the data from 8 WT and 8 KO brains. Mean values ± S.D. (error bars) and S.E.M (boxes) are indicated (*n* = 8; ** *p* < 0.01; * *p* < 0.05, unpaired *t*-test, two-tailed).

In cultured primary E16 cortical cells from the HMGB1 KO, both anti-HMGB1 and anti-AMIGO1 immunostaining were negative (Figure 4A, I and II). It confirms that the HMGB1 KO cells did not have any HMGB1 expression endogenously, or its downstream signal AMIGO1 [40]. As expected from the morphological analysis of brain development (see above), when compared to the WT cells the HMGB1 KO cells showed much lower staining by the neuron-specific anti-NeuN and by anti-GFAP that detects mainly astrocytes (Figure 4A, III). Furthermore, the HMGB1 KO embryos had much less differentiating neurons (anti-Tuj1) in the cortical plate (CP) and the intermediate zone (IZ) supporting the neurogenesis defect in the dorsal telencephalon of the E16 HMGB1 KO (Figure 4A, IV and V & Figure 4B). Furthermore, the HMGB1 KO embryos had more anti-Tuj1 staining in the ventricular zone (VZ) than the WT embryos (Figure 4B), which suggested a migration defect of neurons from the ventricular zone to more superficial parts of the brain. Taken together, the HMGB1 KO mice display defective neuronal differentiation and migration in the developing cortex.

Recent reports have found that the glia precursors in mouse embryos start showing up in developing forebrain from around E14 [43,44]. We measured the glial cell populations in the E14.5 mouse cortical cells by flow cytometry analysis to investigate the gliogenesis and differentiation in the HMGB1 KO brains (Figure 4C). In the WT mouse brain, microglia is over 1.1% of the total cell population, oligodendrocytes about 0.6%, and astrocytes over 6% of the total cell population (Figure 4D). Microglia was somewhat reduced, and a remarkable reduction was seen in the numbers of oligodendrocytes and astrocytes (Figure 4D). Taken together, immunostaining and fluorescence-activated cell sorting (FACS) results have both clearly demonstrated that the HMGB1 KO mouse has a significant reduction of differentiated neural cell populations.

### 3.4. HMGB1 Regulates CXCL12/CXCR4 Expression in the Neuronal Progenitors

Similar to HMGB1, CXCL12 and CXCR4 are broadly expressed in the progenitors of developing brain [29,45,46]. However, the possible role of HMGB1 in regulating CXCL12/CXCR4 signaling in the nervous system has not been explored.

**Figure 5:**
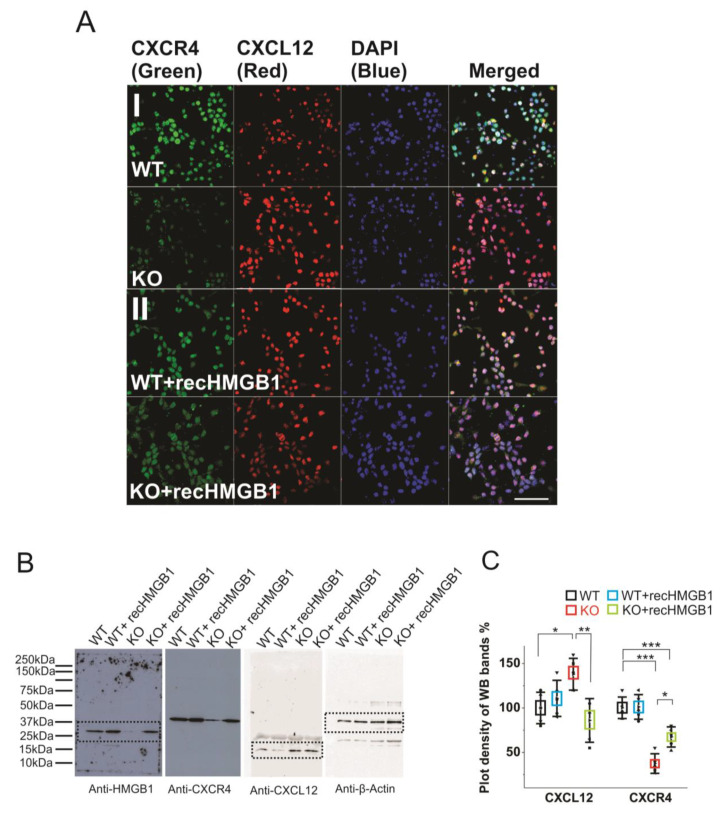
Expression of CXCR4 and CXCL12 in cultured HMGB1 KO and WT brain cells. Cells from cortical cultures of five KO and WT E16 brains were collected and cultured for immunostaining and Western blotting analysis. (**A**) Anti-CXCR4 and Anti-CXCL12 staining of E16 cortical neurons cultured for two days. (I) WT and HMGB1 KO cortical neuronal cells. (II) Cultures with added recombinant HMGB1 (recHMGB1;10 μg. Scale bars indicate 50 μm. All images are confocal z-stack max projections of 10 μm. (**B**) Western blotting of E16 neuronal cell samples cultured for two days. The same sample has been used for all four antibodies in the Western blotting experiment. On lanes with multiple bands the relevant areas have been encircled with rectangles. (**C**) Plot-density analysis of Western blotting bands of the cultured cell samples. Three repeats were applied with the cell samples collected from five KO and five WT brains. CXCL12 in the KO is significantly higher than in the other groups. CXCR4 in the KO and in the KO + recHMGB1 is significantly lower than in the WT, but CXCR4 in the KO + recHMGB1 is significantly higher than in the KO. Mean values ± S.D. (error bars) and SEM (boxes) are indicated (*n* = 5; *** *p* < 0.001; ** *p* < 0.01; * *p* < 0.05, unpaired *t*-test, two-tailed).

Firstly, we have evaluated the CXCR4 and CXCL12 expression levels in primary cortical neuronal cultures derived from the WT and the HMGB1 KO E16 embryos while using immunofluorescence stainings. The WT cells showed much higher CXCR4 expression than the HMGB1 KO cells, while the HMGB1 KO cells expressed much higher levels of CXCL12 than the WT cells (Figure 5AI). After adding the recombinant HMGB1 protein, the HMGB1 KO cells showed increased CXCR4 expression and decreased CXCL12 expression. In contrast, the WT cells did not show much change of CXCR4 and CXCL12 expression after adding the recombinant HMGB1 protein (Figure 5AII).

Furthermore, we have quantified the protein expressions in the brain cell cultures by western blotting (Figure 5B). The plot density analyses clearly showed that the CXCR4 in the HMGB1 KO cells is only about 40% of that in the WT cells, while CXCL12 in the HMGB1 KO cells is over 30% more than in the WT cells (Figure 5C). Furthermore, in the HMGB1 KO cells with recombinant HMGB1 protein, CXCR4 is increased to over 60% of the WT level and CXCL12 has been reduced to almost a normal level (~ 90% of the level in the WT cells). Different from the KO cells, the WT cells did not show any significant difference in CXCR4 and CXCL12 expression with or without extra recombinant HMGB1 (Figure 5C).

**Figure 6:**
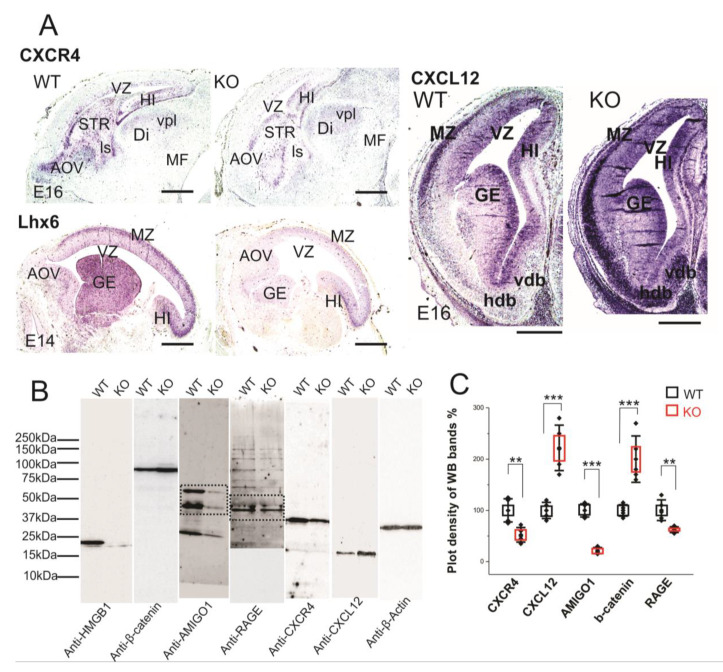
Expression of CXCR4 and CXCL12 in developing forebrain. (**A**) CXCR4 (E16 sagittal section), Lhx6 (E14 sagittal section) and CXCL12 (E16 coronal section) in situ hybridization. The HMGB1 KO mice showed decreased expression of CXCR4 and Lhx6, but increased CXCL12 in the developing cortex compared to the WT mice. Scale bars indicate 1mm. Abbreviations: AOV, ventral anterior olfactory area; Di, diencephalon; GE, ganglionic eminence; Hdb, horizontal nucleus of diagonal band of Broca; HI, hippocampus; ls, lateral septal nucleus; MF; mesencephalic flexure; MZ, Marginal zone; STR, striatum; Vdb, vertical nucleus of diagonal band of Broca; vpl, ventral posterior thalamic nucleus lateral; VZ, Ventricular zone. (**B**) Western blotting of E16 mouse brain samples. The same pair of the WT and KO samples has been used for the Western blotting for each antibody in one experiment. The experiment has been repeated with six pairs of the WT and KO E16 samples. On lanes with multiple bands the relevant areas have been encircled by rectangles. (**C**) Plot-density analysis of Western blotting bands of E16 brain samples. The HMGB1 KO mice showed significantly less expression of CXCR4, AMIGO1and RAGE than the WT mice. In contrast, the HMGB1 KO mice expressed much more CXCL12 and β-Catenin than the WT mice. Statistics of plot-density analyses has been obtained by six repeats. Mean values ± S.D. (error bars) and S.E.M (boxes) are indicated (*n* = 6; *** *p* < 0.001; ** *p* < 0.01; unpaired *t*-test, two-tailed).

We have applied CXCR4 and CXCL12 in situ hybridization on paraffin sections of E16 embryos in order to study the expressions in the brain tissue (Figure 6A). The HMGB1 KO showed significantly lower CXCR4 expression than the WT in the forebrain area, especially in the anterior olfactory area, striatum (STR), and hippocampus (Figure 6A). In contrast, the HMGB1 KO mouse had significantly higher CXCL12 expression than the WT in the forebrain, which was clearly shown by the more intense staining at marginal zone (MZ), ganglionic eminence (GE), and nucleus horizontal diagonal band (hdb) regions (Figure 6A). Furthermore, the western blotting of E16 cortical samples showed that CXCR4 in the HMGB1 KO was significantly decreased, to lower than 50% of the WT level, and CXCL12 in the HMGB1 KO was increased to more than two-fold of the WT level (Figure 6B,C).

CXCL12/CXCR4 signaling has been reported to be crucial for cortical interneuron migration and distribution in the developing neocortex [28,47,48]. LIM/homeodomain Lhx6 is the transcription factor that is preferentially expressed in the cortical interneurons and it is essential for their migration [49]. We therefore applied Lhx6 in situ hybridization on the sections of E14 embryos. The HMGB1 KO embryos demonstrated significantly lower Lhx6 expression in the MZ and GE than the WT in the developing forebrain (Figure 6A). Therefore, it appears that Lhx6 is involved in the regulation of CXCL12/CXCR4 expression in the interneurons of the embryonic brain.

Western blotting showed that RAGE in the HMGB1 KO brain samples was less than 60% of the WT control (Figure 6C). Recent studies in immune cells have shown that HMGB1/RAGE signaling increases the transcription of chemokine receptors to enhance chemotaxis [13,50]. Thus, defective HMGB1/RAGE signaling elucidates the disrupted CXCL12 and CXCR4 expression in the HMGB1 KO brain.

Taken together, both in vivo and in vitro analyses thus show that HMGB1 depletion in the embryonic brain upregulates CXCL12, which consequently downregulates CXCR4.

### 3.5. HMGB1 Depletion Alters Neurodevelopmental Transcription Factors Hierarchy in Developing Forebrain

The vertebrate forebrain development follows a highly ordered stereotypical pattern under the complex genomic regulatory mechanism [51]. In the early development, the anterior-posterior WNT gradient is one key factor regulating the anterior forebrain development [52]. We previously reported that HMGB1 knockdown in zebrafish embryo causes defective forebrain development due to highly increased Wnt/β-catenin expression [14]. Therefore, we first applied western blotting of E16 cortical tissues with anti-β-catenin antibody and found that the E16 HMGB1 KO mouse had over two-fold Wnt/β-catenin expression as compared to the WT (Figure 6B,C). Western blotting results also showed the down-regulation of AMIGO1 expression in the HMGB1 KO (~20% of the WT level), as in the neuronal cultures (Figure 4AII).

**Figure 7:**
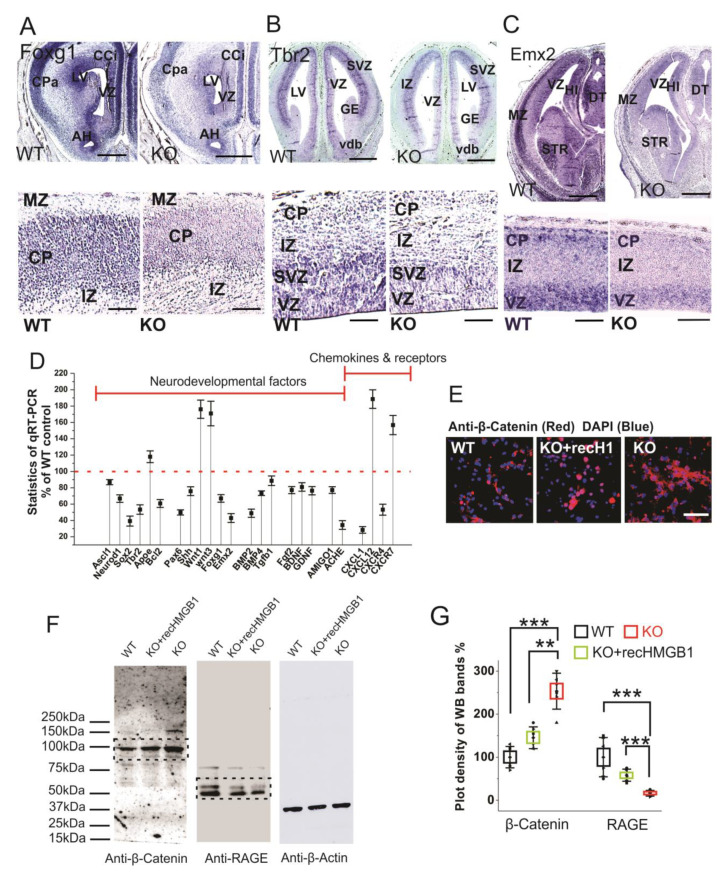
Expressions of neurodevelopmental factors in mouse dorsal telencephalon. (**A**) In situ hybridization of Foxg1 with E16 coronal sections. The higher resolution view in the lower panel clearly showed decreased Foxg1 expression in the CP of HMGB1 KO. The HMGB1 KO mouse also showed shrunken VZ and LV when compared to the WT. Scale bars indicate 1 mm in upper panel, and 200 μm, in the lower panel. (**B**) In situ hybridization of Tbr2 with E14 coronal sections and E16 saggital section (lower panel with higher resolution). The HMGB1 KO showed much less Tbr2 positive cells in the ventricular zone than WT. Scale bars indicate 1 mm in the upper panel and 100 μm in the lower panel. (**C**) In situ hybridization of Emx2 in the E16 coronal sections and E16 sagittal sections (lower panel with higher resolution). The coronal sections show decreased Emx2 expression in the prenatal cortex of the HMGB1 KO mouse when compared to the WT. Scale bar indicates 1.5 mm. The sagittal sections observed under higher resolution showed less Emx2 positive cells in the ventricular zone (VZ) and cortical plate (CP) in the HMGB1 KO as compared to the WT. Scale bar indicates 200 µm, Abbreviations:AH, Anterior horn; CCi, Cingulate cortex; CPa, Parietal cortex; DT, Dorsal thalamus; GE, Ganglionic eminence; HI, Hippocampus; IZ, Intermediate zone; LV, Lateral ventricle; MZ, Marginal zone; Vdb, Vertical nucleus of diagonal band of Broca; STR, Striatum; VZ, Ventricular zone. (**D**) Expression of brain developmental factors detected by qRT-PCR. qRT-PCR has been repeated five times with the samples from five WT and five KO mice. Each sample has been independently tested at least three times. Mean values ± S.E.M (error bars) are indicated (*n* = 5). (**E**) Anti-β-Catenin immunostaining of E16 cortical neurons cultured for two days. Cell nuclei were stained with DAPI (blue). Scale bar indicates 50 µm. (**F**) Anti-β-Catenin and Anti-RAGE Western blotting of E16 neuronal cell samples cultured for 2 days. On lanes with multiple bands the relevant areas are encircled by rectangles. (**G**) Plot-density analysis of Western blotting bands of cultured cell samples. The experiment was repeated with the cell samples collected from six KO and six WT brains. Mean values ± S.D. (error bars) and S.E.M are indicated (*n* = 6; *** *p* < 0.001; ** *p* < 0.01; unpaired *t*-test, two-tailed).

Foxg1 is a conserved factor regulating Wnt in vertebrate CNS development, and it plays a key role in neurogenesis and neuronal fate in the forebrain [53,54]. Foxg1 has been found to promote neurogenesis and inhibit neuronal differentiation in developing forebrain by the transcriptional repression of Wnt ligands [54]. We carried out Foxg1 in situ hybridization with E16 sections due to the increased Wnt signaling detected in the HMGB1 KO embryos. The HMGB1 KO mice showed much lower Foxg1 level along the ventricular layer and the cortical plate than the WT (Figure 7A). Foxg1 has been proven to be an inhibitor of Wnt signaling in developing forebrain as the downstream signal of sonic hedgehog (Shh) [55]. Thus, the decreased Foxg1 appears to be the reason for increased Wnt expression in the HMGB1 KO.

Similar to Foxg1, Tbr2 and Emx2 are two other essential progenitor markers that area expressed in the dorsal telencephalon [56,57]. Tbr2 in situ hybridization showed that the HMGB1 KO mouse had significantly reduced Tbr2 expression in the ventricular zone (VZ) of the dorsal telencephalon (Figure 7B), which likely underscores neurogenesis/proliferation defects shown by BrdU staining and primary neuronal culture of the E16 prenatal cortex (see above). Furthermore, Emx2 in situ hybridization confirms the neurogenesis defects in the developing forebrain of the HMGB1 KO. In the HMGB1 KO E16 dorsal telencephalon, Emx2 expression decreased significantly as compared to the WT control. The HMGB1 KO clearly showed much less Emx2 expression in the marginal zone (MZ), ventricular zone (VZ) layer, and striatum (STR) than the WT control (Figure 7C). The decrease of Foxg1, Tbr2, and Emx2 in the HMGB1 KO forebrain is in agreement with the attenuated neurogenesis during development.

In addition, we have systematically investigated the expression of developmental transcriptional factors and other relevant genes that are involved in neurogenesis and differentiation in the developing brain by qRT-PCR (Figure 7D). When compared with the WT controls, the E16 embryonic brain of the HMGB1 KO showed decreased expression of several neurogenesis factors, such as Ascl1, Neurod1, Sox2, Tbr2, and Bcl2. The HMGB1 KO also had significantly decreased expression of the developmental factors Pax6, Shh, Foxg1, and Emx2. Coincidently, the expression of the differentiation factors BMP2, BMP4, and Tgfβ1 in the HMGB1 KO embryos were downregulated. Not surprisingly, the HMGB1 KO displayed approximately 20% lower expression of neuronal growth factors Fgf2, BDNF, and GDNF. Interestingly, the expression of the synaptogenesis factor Ache was decreased by about 70% in the HMGB1 KO embryo as compared to the WT control. In contrast, the level of the apoptotic signals indicator ApoE was 20% higher in the HMGB1 KO than in the WT. The Wnt1 and Wnt3 levels in the HMGB1 KO were about 80% higher than in the WT controls. This confirmed the increased Wnt/β-catenin signaling in the HMGB1 KO developing brain. The results of anti-β-Catenin antibody immunostaining and western blotting with E16 cortical neuronal cultures clearly showed that Wnt/β-catenin expression was significantly elevated in HMGB1 KO cells (Figure 7E,F). The HMGB1 KO cells that were supplied with recombinant HMGB1 expressed much less Wnt/β -catenin than the KO, but still around 20% more than the WT (Figure 7G). In addition, we detected strongly decreased RAGE expression in the HMGB1 KO cells, which was significantly upregulated by supplying with recombinant HMGB1 (Figure 7F,G). The reversal of the changes in the KO cells by recombinant HMGB1 confirms that HMGB1 can regulate Wnt/β-catenin and RAGE signaling, and the effects in the KO cells cannot be explained by irreversible nonspecific effects.

Furthermore, the qRT-PCR analysis showed that the HMGB1 KO has decreased CXCL1 expression (about 70% less) and increased CXCL12 expression (about 100% more) (Figure 7D). The HMGB1 KO mouse also showed decreased CXCR4 (50% less) and increased CXCR7 (60% more) when compared with the WT controls. Thus, the qRT-PCR analysis confirmed that HMGB1 depletion significantly alters chemokine signaling pathways in the embryonic brain shown by immunostaining and Western blotting experiments (see above, Figure 5).

Taken together, the remarkable changes of gene expressions underscore the observed morphological defects in the HMGB1 KO embryonic brain. Our results show that HMGB1 is a conserved factor crucial for neurogenesis and forebrain development.

## 4. Discussion

### 4.1. HMGB1 Is Crucial for the Neural Stem Cells/Progenitor Cells in the Developing Brain

Based on in vivo and in vitro results, the HMGB1 KO stem cells/progenitor cells showed significantly down-regulated proliferation and up-regulated apoptosis as compared to the WT cells (Figure 1 and Figure 2). Previously, we have found that knocking down HMGB1 increased apoptosis dramatically in zebrafish developing forebrain [14]. The mechanism by which HMGB1 regulates neural progenitor proliferation, differentiation and survival is not fully understood. The increased apoptosis in the HMGB1 KO mouse embryonic brain may be due to the interruption of cytosolic HMGB1/p53 signaling in neuronal progenitors. As a nuclear non-histone protein, HMGB1 is known to facilate p53 binding with DNA in order to promote the autophagy upon inflammatory response [58]. It has been reported that HMGB1 KO embryonic fibroblasts showed more p53 cytosolic localization, which inhibited autophagy and induced apoptosis [59]. In addition, the overexpression of Wnt/β-catenin can also induce the accumulation of p53 protein in the cancer cell cultures [60]. The excessive Wnt/β-catenin expression in the HMGB1 KO mice may be the key reason of the induced neuronal apoptosis and reduced proliferation [61].

As a well-known extracellular ligand of RAGE, HMGB1 binding to RAGE is known to enhance RAGE expression [62]. Therefore, the HMGB1 KO mice had reduced RAGE expression. HMGB1 has been reported to downregulate apoptosis in tumor cells by binding to RAGE [63]. The HMGB1/RAGE signaling may be required for eliminating apoptosis in neuronal progenitors, as in other types of cells. This inference is supported by our finding that the recombinant HMGB1 can diminish the apoptosis of neuronal cultures from the HMGB1 KO mice. Other similar works found that extracellular HMGB1 can act extrinsically on its membrane receptor TLR-4 to facilitate the signaling of pro-survival factors in neuroblastoma [64], and HMGB1/TLR-4 signaling would be activated to inhibit cell apoptosis in cortex of rats with brain injury [65]. Future work would be required to address the question of what extracellular receptors are essential in neural progenitors. However, the extracellular HMGB1 receptors, RAGE and/or TLR-4 may not be essential in HMG1-dependent proliferation, since adding recombinant HMGB1 to the extracellular space did not restore proliferation in the HMGB1 knockout neuronal cultures. It appears that nuclear interactions of HMGB1 may be required for the proliferation of neuronal progenitors.

We have also noticed that HMGB1 depletion induced significant downregulation of neuronal differentiation in developing brain. The FACS results clearly showed that the population of three types of glia cells, astrocytes, oligodendrocytes, and microglia, were all decreased in the E16 brain of the HMGB1 KO when compared to the WT. Different types of glia cells showed different changes in the HMGB1 KO. The population of oligodendrocytes decreased most, and microglia decreased least. Notably, HMGB1 plays a critical role in promoting RAGE-dependent neuronal differentiation in cultured neural progenitor cells and neuroblastoma cells [8,66,67]. HMGB1 has also been reported to be able to promote neuronal differentiation of hippocampal neural progenitor cells via the activation of the RAGE/NF-κB axis [68]. Our data suggest that HMGB1 is required for maintaining neural differentiation through RAGE signaling in the developing CNS.

The grossly decreased oligodendrocyte population in the HMGB1 KO mouse embryonic brain may be caused by the elevated Wnt/β-catenin signaling in vivo. It has been previously shown that the enhanced Wnt/β-catenin signaling inhibits the generation of oligodendrocyte progenitors before gliogenesis, and postpones the maturation of oligodendrocyte precursors [69].

In addition to aberrant Wnt/β-catenin signaling, the reduced differentiation and gliogenesis in the HMGB1 KO may also be due to the significantly decreased AMIGO1. As the member of LRRIG proteins, AMIGO1 expression has been found to be induced by HMGB1 in neuronal cells, and it can facilitate neurite outgrowth and neural tract formation in the developing brain [40,41]. In addition to neurons, AMIGO1 is widely expressed in astrocytes and oligodendrocytes [70]. Studies in vitro have shown that AMIGO1 depletion downregulates the function of the voltage-gated potassium channel (Kv2), which can directly inhibit the proliferation of astrocytes [71,72].

We recently reported that HMGB4, a new member in the family of HMGB proteins, regulates the expression of an oligodendrocyte marker and other neuronal differentiation marker genes [42]. HMGB1 may share a similar mechanism as HMGB4 in regulating neuronal differentiation.

### 4.2. HMGB1 Regulates Brain Development by Wide Interaction With Essential Developmental Transcription Factors

Vertebrate brain structures are largely conserved across species. Importantly, the forebrain is responsible for higher-order functions in mammals. Many neurodevelopmental disorders are due to genetic defects affecting forebrain development [73]. The HMGB1 KO mouse embryos showed massive deficiency in brain development. Similar defective CNS development has also been observed in HMGB1 knockdown in zebrafish embryos [14].

The development of the embryonic forebrain relies on stereotyped expression of a series of transcription factors that are involved in BMP, TGFβ, and Wnt/β-catenin signaling pathways [51,74]. By both qRT-PCR and Western blotting, we have found elevation of Wnt/β-catenin signaling in the E16 HMGB1 KO embryonic brains. The increase of Wnt/β-catenin signaling is likely of significance, as the overactivation of Wnt/β-catenin signaling would suppress embryonic forebrain development [75]. Previous reports showed that forebrain defects in the HMGB1 knockdown zebrafish morphants were also accompanied with the elevation of Wnt signaling [14]. The involvement of HMGB1 in Wnt signaling is also found in Caenorhabditis elegans [76]. These findings show that HMGB1 has a conserved function in regulating Wnt/β-catenin signaling in the developing CNS across species.

HMGB1 is the most abundant nuclear nonhistone protein that was previously considered solely as an architectural protein supporting the chromatin structure by nonspecific binding to DNA and facilitating DNA repair [77]. However, HMGB1 has been shown to facilitate transcription activation by interacting with other nuclear transcription factors [42,78,79,80,81]. It is not surprising to find that HMGB1 depletion induced the aberrant expression of Emx2, Foxg1, and Tbr2, which likely underlies the significantly reduced neurogenesis and differentiation in the developing forebrain. The qRT-PCR results confirmed that the important neurogenesis factors Ascl1, Bcl2, Neurod1, Sox2, and Tbr2 were significantly decreased. The developmental transcription factors that are required for anterior forebrain development, Emx2, Foxg1, Pax6, and Shh, are significantly downregulated in the HMGB1 KO mouse. The HMGB1 KO mouse also has a lower expression of BMP and TGFβ cytokines, which suggests that the development of the HMGB1 KO mouse might be delayed. This might also be one reason of the smaller size of the HMGB1 KO embryos. Decreases of the growth factors BDNF, Fgf2, and GDNF should contribute to the defects of brain development in the HMGB1 KO mouse. The decreased expression of AMIGO1 and ACHE, which are important for the formation of neuronal connections in differentiated cells, coincide with the defects of differentiation in the HMGB1 KO mouse. It is worth noting that HMGB1 depletion did not change expression of Notch, the bHLH factors Hes1 and Olig2 (data not shown). This indicates that HMGB1 may not interact with Notch signaling directly in early developing brain.

### 4.3. HMGB1 Signaling Through CXCL12/CXCR4 in Developing Brain

Our data clearly showed that the HMGB1 KO embryos had significantly more CXCL12, but much less CXCR4 than the WT in the developing CNS. HMGB1-CXCL12 heterocomplex and its receptor CXCR4 have been found to be a key alarmin of inflammation response in the immune system [82].

CXCL12 and CXCR4 have been recently found to be widely involved in brain development by guiding neuronal precursor migration and maintaining the neuronal progenitor pool in embryonic brain, in addition to their crucial role in the innate immune system [23,83,84]. Moreover, HMGB1-CXCL12 chemotaxis can act through CXCR4 to activate stem cells to accelerate tissue regeneration [85,86]. By immunostaining of primary cortical neuron cultures, we showed that recombinant HMGB1 added in the cell cultures significantly alleviated the aberrant CXCL12/CXCR4 expression in the HMGB1 KO cells when compared to the WT cells. Our results suggest that intrinsic HMGB1 directly regulates CXCL12/CXCR4 signaling in the neuronal cells, and extrinsic HMGB1 can affect CXCL12/CXCR4 signaling in the cells lacking the intrinsic HMGB1 regulation.

Furthermore, we found that CXCR7, another receptor of CXCL12, was significantly upregulated in the HMGB1 KO embryos. CXCR7 can regulate CXCL12 availability by acting as a scavenger, and increased CXCR7 would induce the excessive CXCL12-mediated CXCR4 activation and degradation [24,87]. The disrupted expression of CXCR4 and CXCR7 in the HMGB1 KO embryonic brain suggests that HMGB1 is essential for balancing the CXCL12 signal through CXCR4 and CXCR7 in the developing CNS. CXCR4 and CXCR7 have been proven to be crucial for the differentiation and migration of intermediate progenitor cells [47,88]. The transcription factor Lhx6 is mainly expressed in interneurons and it controls their migration. The HMGB1 KO embryos showed decreased Lhx6 expressing interneurons in the developing cortex suggesting a migration defect.

The dramatically increased Wnt/β-catenin signaling in the HMGB1 KO may also rely on the elevated CXCL12, since CXCL12 can induce the accumulation of β-catenin in neuronal progenitors [89]. We suggest that the disrupted CXCL12 signaling through the CXCR4/CXCR7 receptors contributes to the defects seen in the developing CNS of the HMGB1 KO embryos.

As a difference from the CXCL12 expression, we found that CXCL1 expression is significantly decreased in the HMGB1 KO embryos, as shown by qRT-PCR. This suggests that HMGB1 ablation results in specific CXCL12 elevation instead of a general activation of chemokine expression. CXCL1 is known to be one of the key regulators of neuronal progenitor proliferation and differentiation [90]. The downregulation of CXCL1 is also likely to contribute to the decreased neurogenesis and differentiation in the HMGB1 KO embryos.

### 4.4. Is the HMGB1-Dependent Neurogenesis Required for Adult Plasticity Underlying Memory and Learning?

Plasticity and regeneration in the adult brain frequently recapitulate developmental mechanisms. For example, the functional brain plasticity that is required for memory and learning depends on adult neurogenesis from stem cells in the hippocampus [91,92]. It is clear that HMGB1 displays a massive expression in embryonic brain as compared to the adult brain [3], but a high local HMGB1 expression remains in hippocampus, olfactory bulb, and ventricular cell layers in the adult brain [18,93]. Most intriguingly, the expression pattern in the adult corresponds to the brain areas that are still capable of neurogenesis, suggesting a role for HMGB1 in neural stem cells/progenitor cells as in the developing brain.

Combining the current data that HMGB1 regulate neurogenesis in the embryonic brain, localizes to neurogenic areas in the adult and that neurogenesis in the adult hippocampus is required for memory and learning, raises the question whether HMGB1 is involved in the regulation of cognitive functions. The knockout mice used in the current study survive only shortly after birth, and do not therefore allow for studies on memory and learning or other cognitive functions. Interestingly, studies on HMGB1 in traumatic brain injury using regulated global knockout in adult mice have very recently shown that the HMGB1 deficient mice display blunted memory and learning, even without any trauma [94]. No explanation has been available for this intriguing finding. Taken together, we suggest that HMGB1-dependent neurogenesis from stem cells/progenitor cells in the hippocampal regions of the adult, such as the dentate gyrus, is required for functional plasticity underlying memory and learning.

Furthermore, a recent report has identified HMGB1 depletion as a candidate causing intellectual disability and microcephaly in human [95]. Gene defects causing an intellectual disability are often localized to genes that regulate brain plasticity [95,96], which would be compatible with the role of HMGB1 in brain plasticity and as a candidate gene linked to intellectual disability. The current findings that HMGB1 depletion causes a disrupted expression of several genes that are required for neurogenesis and neuronal migration support the finding that HMGB1 depletion during embryonic development causes microcephaly and intellectual disability.

## 5. Concluding Remarks

Our study uncovers the critical role of HMGB1 in mouse forebrain development. The HMGB1 KO mouse showed disrupted expression of several neurodevelopmental factors regulating neurogenesis. Together with previous reports, we confirmed that HMGB1 is crucial in the CNS development across species and may, in addition, regulate plasticity-related functions of the adult brain, which deserves further studies. Furthermore, our data have evidenced that the HMGB1-CXCL12/CXCR4 signaling found in the innate immune system also exists in the mouse developing nervous system. The universal HMGB1 regulation of CXCL12/CXCR4 signaling in both the immune system and the central nervous system suggests that HMGB1 is a crucial messenger of neuro-immune crosstalk. Future studies with conditional HMGB1 KO mouse model would help us to shed light on the interactions and signal transduction between the immune system and the nervous system.
